# Comparison of Post-Exercise Hypotension Responses in Paralympic Powerlifting Athletes after Completing Two Bench Press Training Intensities

**DOI:** 10.3390/medicina56040156

**Published:** 2020-04-01

**Authors:** Ângelo de Almeida Paz, Felipe José Aidar, Dihogo Gama de Matos, Raphael Fabrício de Souza, Marzo Edir da Silva-Grigoletto, Roland van den Tillaar, Rodrigo Ramirez-Campillo, Fábio Yuzo Nakamura, Manoel da Cunha Costa, Albená Nunes-Silva, Anselmo de Athayde Costa e Silva, Anderson Carlos Marçal, Victor Machado Reis

**Affiliations:** 1 Department of Physical Education, Federal University of Sergipe (UFS), 49100-000 São Cristovão, Sergipe, Brazil; angelo-paz@uol.com.br (Â.d.A.P.); pit_researcher@yahoo.es (M.E.d.S.-G.); 2 Department of Physical Education, Tiradentes University (UNIT), 49100-000 Aracaju, Sergipe, Brazil; 3 Group of Studies and Research of Performance, Sport, Health and Paralympic Sports (GEPEPS), the Federal University of Sergipe (UFS), 49100-000 São Cristovão, Sergipe, Brazil; dihogogmc@hotmail.com (D.G.d.M.); raphaelctba@hotmail.com (R.F.d.S.); acmarcal@yahoo.com.br (A.C.M.); 4 Program of Physiological Science, Federal University of Sergipe (UFS), 49100-000 São Cristovão, Sergipe, Brazil; 5 Institute of Parasitology, McGill University, Montreal, QC H3A 0G4, Canada; 6 Scientific Sport Association, 14001–14014 Cordoba, Spain; 7 Department of Sport Sciences and Physical Education, Nord University, 1490 8049 Levanger, Norway; roland.v.tillaar@nord.no; 8 Department of Physical Activity Sciences, Universidad de Los Lagos, 14104 Osorno, Chile; r.ramirez@ulagos.cl; 9 Department of Physical Education, Federal University of Paraíba-UFPB, 50670-901 João Pessoa, Paraíba, Brazil; fabioy_nakamura@yahoo.com.br; 10 Human Performance Laboratory, Pernambuco State University-(UPE), 50100-010 Recife, Pernambuco, Brazil; mcosta2@gmail.com; 11 Laboratory of Inflammation and Exercise Immunology, Sports Center, Federal University of Ouro Preto, 35400-000 Minas Gerais, Brazil; albenanunes@hotmail.com; 12 Department of Physical Education, Federal University of Pará (UFPA), 66075-110 Belém, Pará, Brazil; anselmocostaesilva@yahoo.com.br; 13 Research Center in Sports Sciences, Health Sciences and Human Development (CIDESD), Trás os Montes and Alto Douro University, 5001-801 Vila Real, Portugal; victormachadoreis@gmail.com

**Keywords:** hypertension, arterial pressure, blood pressure, hypotension, resistance training

## Abstract

*Background and objective*: Post-exercise hypotension, the reduction of blood pressure after a bout of exercise, is of great clinical relevance. Resistance exercise training is considered an important contribution to exercise training programs for hypertensive individuals and athletes. In this context, post-exercise hypotension could be clinically relevant because it would maintain blood pressure of hypertensive individuals transiently at lower levels during day-time intervals, when blood pressure is typically at its highest levels. The aim of this study was to compare the post-exercise cardiovascular effects on Paralympic powerlifting athletes of two typical high-intensity resistance-training sessions, using either five sets of five bench press repetitions at 90% 1 repetition maximum (1RM) or five sets of three bench press repetitions at 95% 1RM. *Materials and Methods*: Ten national-level Paralympic weightlifting athletes (age: 26.1 ± 6.9 years; body mass: 76.8 ± 17.4 kg) completed the two resistance-training sessions, one week apart, in a random order. *Results*: Compared with baseline values, a reduction of 5–9% in systolic blood pressure was observed after 90% and 95% of 1RM at 20–50 min post-exercise. Furthermore, myocardial oxygen volume and double product were only significantly increased immediately after and 5 min post-exercise, while the heart rate was significantly elevated after the resistance training but decreased to baseline level by 50 min after training for both training conditions. *Conclusions*: A hypotensive response can be expected in elite Paralympic powerlifting athletes after typical high-intensity type resistance-training sessions.

## 1. Introduction

It has been previously noted that 12% of Paralympic athletes have cardiovascular abnormalities, and 2% are at high risk of sudden death and showing arrhythmogenic cardiomyopathies, among other cardiac diseases [1]. Paralympic powerlifting is a strength-based sport that has as its only component the adapted bench press, designed for people with some form of physical disability, and its goal is a greater weight lifting capacity [2].

Previous researchers have found that regular resistance training is an important strategy to control systemic blood pressure in both normotensive and hypertensive subjects [3,4]. Among the effects of resistance training on the cardiovascular system, post-exercise hypotension has been studied in hypertensive subjects with clinically relevant implications [5,6]. More recent studies have shown that individuals with large post-exercise hypotension after an exercise session can be predicted to exhibit such post-exercise hypotension even after weeks of training [7].

The mechanisms involved in post-exercise hypotension have been attributed to reduced peripheral vascular resistance, decreased sympathetic activity, diminished systolic volume, and changes in the sensitivity of adrenergic, cardiac, and endothelial factors [8]. The American College of Sports Medicine (ACSM) [9] and the American Heart Association [4] have both stated that resistance training, in association with an aerobic-based exercise program, is an efficient method to prevent, treat, and control arterial hypertension. In addition, only a few studies [10,11] have evaluated the effects of the volume and intensity of resistance training on the magnitude of post-exercise hypotension.

Of particular relevance, the intensity of resistance training may be considered especially important in sport disciplines where high-intensity resistance training is regularly used. Paralympic powerlifting, derived from conventional powerlifting, is a sport discipline that relies on high-intensity resistance training to develop muscle strength. It is a sport based on maximum muscle strength, where athletes attempt to lift the heaviest possible load at one maximum repetition (1RM) in three attempts in squatting, ground lifting, and the bench press [12,13]. Haslam et al. [14] showed that heavier loads lead to larger increases in blood pressure and heart rate. In addition, the exercises utilized in a powerlifting exercise session are composed of movements that can lead to high blood pressure and heart rate values [15,16].

According to the ACSM [9], recommendations of high-intensity resistance training (≥80% of 1RM) have been used by athletes, recreational exercisers, and fitness center practitioners as a strategy to increase muscular strength. However, there is a gap in the literature about the influence of resistance training intensities using loads near 100% of 1RM, which are strategies commonly used by powerlifting athletes. Moreover, there is a paucity of scientific information regarding the effects of high-intensity resistance training on Paralympic powerlifting athletes.

To our knowledge, this is the first study to investigate the post-exercise hypotensive responses in Paralympic powerlifting athletes. Moreover, the present study addresses the question whether high-intensity bench pressing performed by Paralympic athletes also exhibit the typical post-exercise hypotensive response. Therefore, we hypothesized that the high-intensity resistance training used in Paralympic powerlifting training may affect the acute responses of hemodynamic variables, generating a possible hypotensive effect. The aim of this study was to compare the post-exercise hypotensive effects on Paralympic powerlifting athletes of two typical high-intensity resistance-training sessions, using either five sets of five repetitions of the bench press at 90% (90% 1RM) or five sets of three repetitions of the bench press at 95% 1RM, training methods that are in common use. 

## 2. Materials and Methods

### 2.1. Subjects

The participants consisted of 10 male Brazilian Paralympic powerlifting athletes (age: 26.1 ± 6.9 years; body mass: 76.8 ± 17.4 kg; 1RM bench press: 117.4 ± 23.4 kg) with a minimum of 12 months of training (2.5 ± 0.2). All were competitors at the national level and ranked among the top 10 of their respective body-mass categories. All athletes were capable of lifting ≥1.4 times their body mass in the bench press (1.7 ± 0.3). Thus, these athletes are considered to be elite athletes [17].

All participants met the prerequisites of the Brazilian Paralympic Committee to be eligible for this sport [2]. Elite athletes are subjected to anti-doping tests and, therefore, did not use any medication that could influence hemodynamic responses. Four athletes had a spinal cord injury below the eighth thoracic vertebra, two had sequelae due to poliomyelitis, two had a malformation of the lower limbs, and two were paraplegic because of a brain injury. Athletes were not included in the study if they: (i) reported the consumption of illicit substances (e.g., anabolic steroids), (ii) had been previously diagnosed with a cardiac or metabolic disease, or (iii) were involved in any process to induce rapid weight loss at the time of recruitment. The athletes participated voluntarily and signed an informed consent form in accordance with Resolution 466/2012 of the National Commission for Research Ethics (CONEP) of the National Health Council, and the ethical principles of the latest version of the Declaration of Helsinki (and the World Medical Association).This study was approved by the Research Ethics Committee of the Federal University of Sergipe CAEE with approval number 79909917.0.0000.55.46.

### 2.2. Procedures

Athletes completed a baseline measurement session to assess 1RM in the bench press using an official bench (Eleiko, Chicago, IL, USA) approved by the International Paralympic Committee [2] and an IPC Olympic bar (Eleiko, Chicago, IL, USA). The 1RM test was conducted, and each subject started the trials with a weight they believed that they could lift once, using maximum effort. Weight increments were then added until they reached the maximum load that could be lifted once. If the participant could not perform a single repetition, 2.4% to 2.5% was subtracted from the load used in the test [18]. The subjects rested for 3 to 5 min between attempts.

The test was preceded by a warm-up set (10 to 12 repetitions) with approximately 50% of the load to be used for the first attempt of the 1RM test. The testing started two min after the warm-up. The load recorded as 1RM was the one when the individual could complete only one repetition. The form and the adapted technique used in the performance of each attempt was standardized and continuously monitored to ensure the quality of the data.

**Figure 1:**
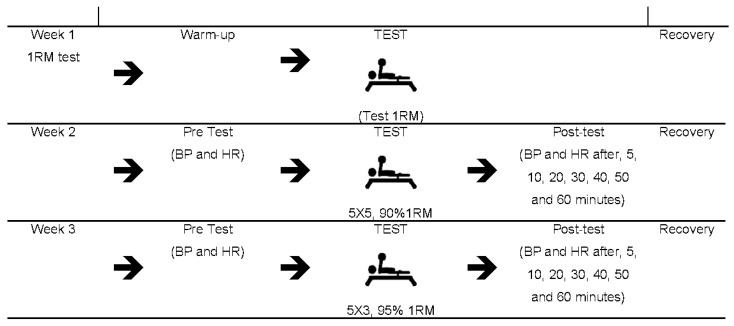
Experimental design of the study. BP: blood pressure, HR: heart rate, 5X5: five sets of 5 repetitions, 5X3: five sets of three repetitions; 1RM: one repetition maximum.

After this, the athletes re-visited the laboratory twice (with one week of rest between visits) to be assessed for resting blood pressure (Tycos, NY, USA) and heart rate (Polar^®^ RS800CX, Kempele, Finland). After 10 min rest to measure baseline values [19], athletes initiated a resistance-training session in a random order, consisting of either five sets of five repetitions of the bench press at 90% of 1RM or five sets of three repetitions of the bench press at 95% of 1RM. After completion of the five sets, blood pressure and heart rate were measured again after 5, 10, 20, 30, 40, 50, and 60 min of rest (Figure 1).

The systolic and diastolic blood pressure, mean arterial blood pressure (mean arterial blood pressure = diastolic + (systolic ‒ diastolic)/3), and heart rate were measured before and after each training session using an automated noninvasive blood pressure monitor (Microlife 3AC1-1PC, Microlife, Widnau, Switzerland). The double product was evaluated according to the following equation: double product = heart rate × systolic blood pressure [20]. All blood pressure measurements were taken using the subject’s left arm. The pre-exercise blood pressure for all subjects did not exceed 160 mm Hg and 100 mm Hg for systolic and diastolic blood pressure, respectively. Measurements were done by having the subjects seated. Automated BP monitor was calibrated according to the manufacturer’s instructions.

The participants were also instructed to avoid the Valsalva maneuver during the entire training session, following the ACSM guidelines [9]. Blood pressure and heart rate were measured in the sitting position (resting) immediately after and at 5, 10, 20, 30, 40, 50, and 60 min after the exercise session, as described above. To estimate myocardial oxygen volume (MVO₂), a mathematical function was used, expressing the result in ml O_2_/100 g ventilations per minute (VE/min), as follows: MVO_2_ = (double product × 0.0014) − 6.37 [21,22].

### 2.3. Resistance Training Sessions

The resistance training sessions consisted of bench press sets, using either 85–90% or 90–95% of 1RM. For the 85–90% of 1RM session, athletes completed five sets of five repetitions, and for the 90–95% of 1RM session, athletes completed five sets of three repetitions, with five min of rest between sets for both protocols [22,23]. The execution of the exercises was standardized according to the competitive rules of the IPC [2]. The measurement was performed by a single evaluator, a technician in Medical Emergencies, Basic level, with 30 years of experience at the Emergency Medical Service of the Fire and Rescue Military Minas Gerais State.

### 2.4. Statistics

The mean and standard deviation values were used to describe the results. To verify the normality of the variables, the Shapiro-Wilk test was used. A two-way analysis of variance 2 (3RM vs. 5RM) × 9 (test time) with repeated measures was used. In cases where the Mauchly test of sphericity indicated that the assumption of sphericity was violated, a Greenhouse-Geisser correction was performed. Holm-Bonferroni’s post-hoc tests were used to compare resistance training intensities. The level of significance was set at *p* < 0.05. Effect size was evaluated with Eta partial squared (η^2^_p_), where 0.01 < η^2^ < 0.06 constituted a small effect, 0.06 < η^2^ < 0.14 a medium effect, and η^2^ > 0.14 a large effect [24]. The statistical analysis was performed using the Statistical Package for Social Science, version 25.0 (SPPS Inc., Chicago, IL, USA).

## 3. Results

**Figure 2:**
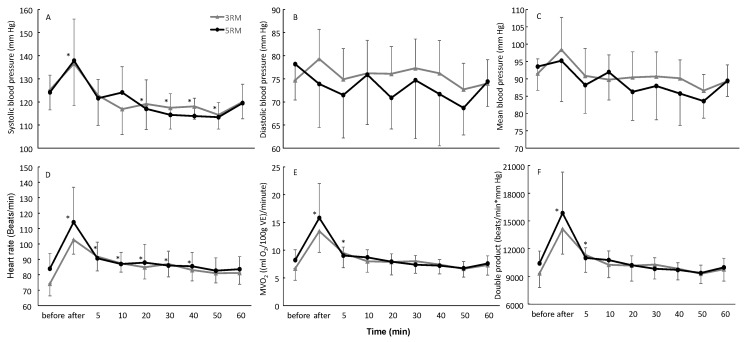
Kinetics of: (**A**) systolic blood pressure, (**B**) diastolic blood pressure, (**C**) mean blood pressure, (**D**) heart rate, (**E**) myocardial oxygen consumption (MVO_2_), and (**F**) double product before and after bench press resistance exercise using either 90% (5RM) or 95% (3RM) of one repetition maximum. * indicates a significant difference with the baseline measurement on a *p* < 0.05 level.

No significant difference was found between the two resistance training sessions for any of the variables (F ≤ 3.9, *p* ≥ 0.082, η^2^_p_ ≤ 0.3). A significant effect of test time was found for all variables (F ≥ 5.1, *p* ≤ 0.005, η^2^_p_ ≥ 0.36) except for diastolic blood pressure (F = 1.4, *p* = 0.256, η^2^_p_ = 0.14). No significant interaction (training*time) was observed (F ≤ 2.3, *p* ≥ 0.12, η^2^_p_ ≤ 0.20). Post-hoc comparisons revealed that systolic blood pressure was significantly increased immediately after both resistance training conditions, followed by a decrease, which significantly declined to under the baseline measurement from 20 to 50 min (−5.8 to 9%) after the resistance training (Figure 2). The myocardial oxygen volume and double product were only significantly increased immediately after and 5 min after the resistance training, while the heart rate was significantly elevated after the resistance training before decreasing to the baseline level by 50 min after training for both conditions (Figure 2).

## 4. Discussion

The aim of this study was to compare the post-exercise hypotensive effects on Paralympic powerlifting athletes of two typical high-intensity resistance-training sessions, using either five sets of five repetitions of the bench press at 90% (90% 1RM) or five sets of three repetitions of the bench press at 95% 1RM. The main findings were that the systolic blood pressure was reduced between 20–50 min post-exercise compared to baseline with no difference between the two conditions. Furthermore, myocardial oxygen volume and double product were significantly increased immediately after and 5 min after the resistance training, while the heart rate was significantly elevated after the resistance training and then it decreased to the baseline level by 50 min after training for both conditions.

Although no significant differences were found between the strength training protocols and any variables, the present study provided some rather curious findings. It was observed that there was a hypotensive hemodynamic response as a consequence of the high-intensity training, regardless of the number of repetitions and sets, highlighting this effect occurred even when the athletes only performed 3 repetitions of the bench press. Consistent with our findings, another study also evaluated athletes working at a high intensity of 1RM with five sets of 2RM in a single powerlifting session, and this was not enough to show hemodynamic overload at the peak of the exercise [22].

There is a growing discussion of the interrelationship between resistance training volume and intensity on post-exercise hypotension [10]. That study [10] found a longer post-exercise hypotension response after 70% of 1RM condition compared with the 80% of 1RM condition. With fewer sets performed to repetition failure at the lowest intensity, there might have been less predominance of the sympathetic nervous system during and after the 70% of 1RM condition [25]. Thus, it seems from this study that the 90% or 95% of 1RM condition involved the right trade-off between less sympathetic nervous system activation to stimulate the longest duration of a reduction in post-exercise blood pressure in Paralympic powerlifting athletes.

Regardless of the intensity used, our findings showed no hemodynamic overload at peak exercise (160 bpm) similar to studies that applied loads greater than 90% of 1 RM during a single weightlifting session [22]. One of the mechanisms that may explain systolic blood pressure dropping below the baseline (verified after 50 min) is a decrease in cardiac output, which in turn is caused by a reduction of the concomitant discharge volume at the same time as an increased heart rate [26].

In addition, studies have also shown that resistance training can improve endothelial nitric oxide synthase biosynthesis and activity, thereby increasing levels of nitric oxide production, which plays a key role in vascular control, mediating blood pressure reduction [27]. Other mechanisms, such as reduced peripheral vascular resistance [7] and arterial stiffness [28], are also mechanisms at stake and concentric cardiac remodeling associated with resistance training is possible.

No change in diastolic pressure was observed immediately after exercise, ensuring safety in the training session. The possible mechanism suggested by Rezk et al. [11] is a decrease in ventricular pre-load, while the peripheral vascular resistance remains unchanged. Thus, there is a decrease in cardiac output and a decrease in blood pressure. The decrease in ventricular pre-load can be influenced by a decrease in venous return caused by a decrease in plasma volume, since during resistance training, blood plasma is pressed into interstitial spaces, decreasing the blood volume [11].

An increase in heart rate, myocardial oxygen volume, and double product after exercise was observed, which is explainable by the fact that high intensities (>80% 1RM) appear to reduce systolic volume-mediated cardiac output [15,29]. The decrease in systolic volume would be compensated for by an increase in heart rate caused by an increase in sympathetic activity and a reduction of the parasympathetic influence on the heart [22].

However, our study has some limitations. Firstly, the mechanisms causing the hypotension were not investigated in the present study. We did not assess peripheral vascular resistance, sympathetic activity, systolic volume, beta-adrenergic receptors, nor endothelial factors. Secondly, the auscultation method was used for assessing blood pressure. This method, while universally used, has some limitations compared to invasive methods, such as intra-arterial catheterization. However, every effort was made to ensure that these measures were obtained in a consistent, reliable, and accurate manner.

## 5. Conclusions

We conclude that the two types of training analyzed resulted in an equal hypotensive response in elite Paralympic powerlifting athletes. Therefore, there is no evidence that Paralympic powerlifting involves cardiovascular stress above those typically found in resistance exercise performed by healthy individuals. The results suggest that Paralympic powerlifting, performed by experienced athletes with diverse physical disabilities and with loads above 85% of 1RM, can be relatively safe.

## References

[1] Pelliccia A., Adami P.E., Quattrini F., Squeo M.R., Verdile L., Spataro A. (2017). Are Olympic athletes free from cardiovascular diseases? Systematic investigation in 2352 participants from Athens 2004 to Sochi 2014. Br. J. Sports Med..

[2] International Paralympic Comite (IPC) Rules. Official Website of IPC Powerlifting. http://www.paralympic.org/powerlifting/about.

[3] Wewege M.A., Thom J.M., Rye K.A., Parmenter B.J. (2018). Aerobic, resistance or combined training: A systematic review and meta-analysis of exercise to reduce cardiovascular risk in adults with metabolic syndrome. Atherosclerosis.

[4] Williams M.A., Haskell W.L., Amsterdam E.A., Bittner V., Franklin B.A., Stewart K.J. (2007). Resistance exercise in individuals with and without cardiovascular disease: 2007 update: A scientific statement from the American Heart Association Council on Clinical Cardiology and Council on Nutrition, Physical Activity, and Metabolism. Circulation.

[5] Duncan M.J., Birch S.L., Oxford S.W. (2014). The effect of exercise intensity on postresistance exercise hypotension in trained men. J. Strength Cond. Res..

[6] Queiroz A.C.C., Sousa J.C.S., Cavalli A.A.P., Silva Junior N.D., Costa L.A.R. (2015). Post-resistance exercise hemodynamic and autonomic responses: Comparison between normotensive and hypertensive men. Scand. J. Med. Sci. Sports.

[7] Brito L.C., Fecchio R.Y., Peçanha T., Andrade-Lima A., Halliwill J.R., Forjaz C.L. (2018). Postexercise hypotension as a clinical tool: A “single brick” in the wall. J. Am. Soc. Hypertens..

[8] Rondon M.U., Alves M.J., Braga A.M., Barretto A.C., Krieger E.M., Negrão C.E. (2002). Postexercise blood pressure reduction in elderly hypertensive patients. J. Am. Coll. Cardiol..

[9] American College of Sports Medicine (2009). American College of Sports Medicine position stand. Progression models in resistance training for healthy adults. Med. Sci. Sports Exerc..

[10] Bentes C.M., Costa P.B., Neto G.R., Costa e Silva G.V., de Salles B.F., Miranda H.L., Novaes J.S. (2015). Hypotensive effects and performance responses between different resistance training intensities and exercise orders in apparently health women. Clin. Phys. Funct. Imag..

[11] Rezk C.C., Marrache R.C., Tinucci T., Mion D., Forjaz C.L. (2006). Post-resistance exercise hypotension, hemodynamics, and heart rate variability: Influence of exercise intensity. Graefe’s Arch. Clin. Exp. Ophthalmol..

[12] João G.A., Lopes Evangelista A., Gomes J.H., Charro M.A., Bocalini D., Cardozo D. (2014). Effect of 16 Weeks of Periodized Resistance Training on Strength Gains of Powerlifting Athletes. J. Exerc. Physiol. Online.

[13] Pritchard H.J., Morton R.H. (2015). Powerlifting: Success and failure at the 2012 Oceania and 2013 classic world championships. J. Aust. Strength Cond..

[14] Haslam D.R., McCartney N., McKelvie R.S., MacDougall J.D. (1988). Direct measurements of arterial blood pressure during formal weightlifting in cardiac patients. J. Cardiopulm. Rehabil. Prev..

[15] Boroujerdi S.S., Rahimi R., Noori S.R. (2009). Effect of high-versus low-intensity resistance training on post-exercise hypotension in male athletes. Int. Sport Med. J..

[16] MacDonald J.R. (2002). Potential causes, mechanisms, and implications of post exercise hypotension. J. Hum. Hypert..

[17] Ball R., Weidman D. (2018). Analysis of USA powerlifting federation data from January 1, 2012–June 11, 2016. J. Strength Cond. Res..

[18] Fleck S.J., Kraemer W. (2014). Designing Resistance Training Programs, 4E.

[19] Bonsu B., Terblanche E. (2016). The training and detraining effect of high-intensity interval training on post-exercise hypotension in young overweight/obese women. Eur. J. Appl. Physiol..

[20] Schutte R., Thijs L., Asayama K., Boggia J., Li Y., Hansen T.W., Jeppesen J. (2013). Double product reflects the predictive power of systolic pressure in the general population: Evidence from 9,937 participants. Am. J. Hypertens..

[21] Aksentijević D., Lewis H.R., Shattock M.J. (2016). Is rate–pressure product of any use in the isolated rat heart? Assessing cardiac ‘effort’ and oxygen consumption in the Langendorff-perfused heart. Exp. Physiol..

[22] João G.A., Bocalini D.S., Rodriguez D., Ceschini F., Martins A., Figueira Junior A. (2017). Powerlifting sessions promote significant post-exercise hypotension. Braz. J. Sports Med..

[23] Austin D., Mann B. (2012). Powerlifting.

[24] Cohen J. (1982). A power primer. Psychol. Bull..

[25] Coelho-Júnior H.J., Irigoyen M.C., de Oliveira Gonçalves I., Câmara N.O.S., Cenedeze M.A., Uchida M.C. (2017). Acute effects of power and resistance exercises on hemodynamic measurements of older women. Clin. Int. Aging.

[26] Terra D.F., Mota M.R., Rabelo H.T., Lima R.M., Ribeiro A.G., Silva F.M.D. (2008). Reduction of arterial pressure and double product at rest after resistance exercise training in elderly hypertensive women. Arq. Bras. Cardiol..

[27] Macedo F.N., Mesquita T.R., Melo V.U., Mota M.M., Silva T.L., Santana M.N. (2016). Increased nitric oxide bioavailability and decreased sympathetic modulation are involved in vascular adjustments induced by low-intensity resistance training. Front. Physiol..

[28] Li Y., Hanssen H., Cordes M., Rossmeissl A., Endes S., Schmidt-Trucksäss A. (2015). Aerobic, resistance and combined exercise training on arterial stiffness in normotensive and hypertensive adults: A review. Eur. J. Sport Sci..

[29] De Sousa E.C., Abrahin O., Ferreira A.L.L., Rodrigues R.P., Alves E.A.C., Vieira R.P. (2017). Resistance training alone reduces systolic and diastolic blood pressure in prehypertensive and hypertensive individuals: Meta-analysis. Hypertens. Res..

